# The effectiveness of colchicine combined with mitomycin C to prolong bleb function in trabeculectomy in rabbits

**DOI:** 10.1371/journal.pone.0213811

**Published:** 2019-03-19

**Authors:** Taiki Kokubun, Kotaro Yamamoto, Kota Sato, Takahiro Akaishi, Atsushi Shimazaki, Masatsugu Nakamura, Yukihiro Shiga, Satoru Tsuda, Kazuko Omodaka, Toru Nakazawa

**Affiliations:** 1 Department of Ophthalmology, Tohoku University Graduate School of Medicine, Sendai, Miyagi, Japan; 2 Collaborative Program for Ophthalmic Drug Discovery, Tohoku University Graduate School of Medicine, Sendai, Miyagi, Japan; 3 Research and Development Division, Santen Pharmaceutical Co. Ltd., Ikoma, Nara, Japan; 4 Department of Retinal Disease Control, Ophthalmology, Tohoku University Graduate School of Medicine, Sendai, Miyagi, Japan; 5 Department of Advanced Ophthalmic Medicine, Tohoku University Graduate School of Medicine, Sendai, Miyagi, Japan; 6 Department of Ophthalmic imaging and information analytics, Tohoku University Graduate School of Medicine, Sendai, Miyagi, Japan; Massachusetts Eye & Ear Infirmary, Harvard Medical School, UNITED STATES

## Abstract

**Purpose:**

To investigate the potential of colchicine to improve bleb function after trabeculectomy.

**Methods:**

To find the maximum usable colchicine concentration, an ocular irritation study was performed with the Draize test at concentrations of 0.001%, 0.01% and 0.1%. Additionally, the synergistic effect of topical colchicine instillation and MMC application to surgical site was evaluated in a rabbit model by measuring changes after trabeculectomy in intraocular pressure (IOP) and bleb morphology score at 3, 7, 14, 21, 28, 35, 42, and 49 days.

**Results:**

Experiments with a rabbit model of trabeculectomy showed that 0.04% MMC plus 0.01% colchicine was more effective than saline and 0.04% MMC alone in maintaining IOP reduction at days 7–49 (P < 0.01 at all time points) and day 49 (P < 0.05), respectively, while 0.04% MMC alone was more effective than saline only at days 7–35 (P < 0.05 at all time points). 0.04% MMC plus 0.01% colchicine and 0.04% MMC alone were more effective than saline at preserving bleb score at days 7–21 and 35–49 (P < 0.05 at all time points) and at days 7–35 (P < 0.05 at all time points), respectively.

**Conclusion:**

Colchicine may be a promising adjuvant for strengthening the effect of MMC and improving the survival of the filtering bleb in trabeculectomy.

## Introduction

Trabeculectomy is one of the most common types of filtration surgery to reduce intraocular pressure (IOP) in glaucoma[1]. This technique involves creating a filtering bleb, through which aqueous humor flows from the anterior chamber into the sub-Tenon’s space and is absorbed into the bleb wall (which consists of Tenon’s tissue). The success of trabeculectomy depends on the postoperative wound healing process in the filtering bleb. During healing, the excessive proliferation of fibroblasts or fibrosis in the subconjunctiva causes tissue scarring, and this scarred tissue cannot adequately absorb the aqueous humor, causing bleb failure[2,3].

Clinically, to prevent fibrosis after trabeculectomy, adjuvants such as mitomycin C (MMC) are used to counteract the proliferative activity of cells[4–6]. Currently, adjuvant MMC is the ‘gold standard’ against which other potential anti-fibrotic therapeutics are compared. However, although the success rate is improved with the use of MMC, bleb failure still occurs in some cases due to strong fibrosis. Increased IOP can then re-occur, necessitating additional treatment[7,8]. In addition, MMC has nonspecific cytotoxic effects that are associated with severe complications, such as blebitis, keratitis, bleb leakage, chronic hypotony and endophthalmitis caused by the un-targeted, generally destructive cellular toxicity of MMC[9–11]. Hence, it is important to identify safer and more broadly targeted anti-fibrotic agents that can maintain bleb function for a longer period and can be used postoperatively in addition to MMC, to help preserve the filtering bleb when it shows signs of failure.

Previous reports have shown that colchicine can suppress migration and proliferation of fibroblasts, in addition to its well-known ability to act as an anti-inflammatory[12,13]. Furthermore, the administration of colchicine can prolong the maintenance of a functional bleb after glaucoma filtration surgery in human subjects by suppressing inflammatory fibrosis[14,15]. However, there are no detailed reports comparing the effectiveness of MMC and colchicine and investigating the effectiveness of colchicine combined with MMC for preserving the filtering bleb.

In the present study, we evaluated the effect of topical colchicine administration in a rabbit model of trabeculectomy. We determined the most appropriate concentration of colchicine to ensure both effectiveness and safety, and investigated the potential of colchicine eye drops as a supplementary postoperative adjuvant in trabeculectomy.

## Materials & methods

### Reagents

Colchicine was purchased from Nacalai Tesque, Inc (Kyoto, Japan). Mitomycin C (MMC; 2-mg injection) was purchased from Kyowa Hakko Kirin Co., Ltd. (Tokyo, Japan). Ketamine hydrochloride (Ketalar for intramuscular injection; 500 mg) was purchased from Daiichi Sankyo Co., Ltd. (Tokyo, Japan). Xylazine hydrochloride (Selactar for injection; 2%) was purchased from Bayer Yakuhin Ltd. (Osaka, Japan). A 0.01% ophthalmic solution of betamethasone sodium phosphate was purchased from Shionogi Co., Ltd. (Osaka, Japan). A 0.5% ophthalmic solution of levofloxacin hydrate and a 0.4% ophthalmic solution of oxybuprocaine were supplied by Santen Pharmaceutical Co., Ltd. (Osaka, Japan).

### Animals

Male Japanese white rabbits, weighing 1.7 to 2.9 kg, were supplied by Kitayama Labes Co., Ltd. (Nagano, Japan). The animals were housed under a 12-hour light/dark cycle and had free access to water and a standard laboratory diet. All animal care and experimental procedures were performed in accordance with the ARVO Statement for the Use of Animals in Ophthalmic and Vision Research and were approved and monitored by the Animal Care and Use Committee of Santen Pharmaceutical Co., Ltd (Approval number: DR120505 and DR120520). After the study was completed, animals were euthanized with an intravenous injection of pentobarbital.

### Ocular irritation

To find the maximum safety colchicine concentration, an ocular irritation study was performed with the Draize test[16]. Twelve male albino rabbits with no existing ocular irritation were used to evaluate each test material; the animals were assigned to one of four groups (n = 3 in each group) in each experiment.

A 50 μL test preparation (containing 0.001%, 0.01% or 0.1% colchicine dissolved in saline) or saline was instilled in one eye. This was repeated every 30 min for 4.5 h (10 times). The ocular response was then scored at 0.5, 1, 2 and 4, and 24 h after the last instillation. Irritation was scored using the Draize method[16]. This method involves weighting and summing six directly observable changes in the eye’s anterior segment: density (A; score: 1–4), area of corneal opacification (B; score: 1–4), severity of iritis (C; score: 1–2), conjunctival redness (D; score: 1–3), edema (E; score: 1–4), and discharge (F; score: 1–3). The mean total score was calculated as (A×B×5)+(C×5)+(D+E+F)×2, ranging from 0 to 110 at each measured time point.

### Surgical procedure

After the induction of general anesthesia, which was performed with an intramuscular injection of ketamine hydrochloride (40 mg/kg) and xylazine hydrochloride (4 mg/kg), trabeculectomy was performed with previously reported methods[17,18]. Briefly, a limbus-based conjunctival flap was prepared, a scleral flap was created, and Medical Quick Absorber (MQA) (Inami Co, Ltd. Tokyo, Japan) saturated with 0.04% MMC solution or saline was placed under the conjunctiva and over the scleral flap site for 5 minutes. The area was irrigated with 120 ml of 0.9% sodium chloride solution (Otsuka Pharmaceutical Factory Inc., Tokushima, Japan). Sclerotomy was performed under the scleral flap; a Kelly Descemet’s membrane punch (M.E. Technica, Tokyo Japan) was used to form a scleral tunnel opening into the aqueous chamber. The scleral flap was not sutured, but the conjunctiva was sutured with 10–0 sutures. Topical 0.01% betamethasone sodium phosphate and 0.5% levofloxacin hydrate ophthalmic solution were administered three or four times daily (according to specific experimental protocols) for the first five days after surgery. The right eye underwent this procedure in all rabbits, while the other eye was left untreated as a control. During this study, no perioperative complications, such as inadvertent penetration to the anterior chamber or aqueous drainage, were observed in any of the eyes.

### Treatment regimens

To compare colchicine and MMC, experiment A was performed, which included 8 rabbits. The animals received either 0.04% MMC or 0.01% colchicine (each n = 4). To compare colchicine combined with MMC vs. MMC alone, experiment B was performed, which included 20 rabbits assigned to receive saline (n = 4), 0.04% MMC (n = 8), or 0.04% MMC plus 0.01% colchicine (n = 8). IOP values were confirmed to be approximately the same in all groups before trabeculectomy.

In experiments A and B, the 0.04% MMC groups were treated with MQA saturated with 0.04% MMC, applied under the conjunctiva and over the scleral flap site for 5 minutes during glaucoma filtration surgery. Saline (50 μL) was then instilled 4 times daily in experiment A and 3 times daily in experiment B. In experiment A, the 0.01% colchicine group received saline under the conjunctiva and over the scleral flap site during glaucoma filtration surgery. After surgery, colchicine (0.01%) eye drops were applied 4 times daily. In experiment B, the control group was treated with MQA saturated with saline, applied under the conjunctiva and over the scleral flap site for 5 minutes during glaucoma filtration surgery, and saline was instilled 3 times daily. The 0.04% MMC plus 0.01% colchicine group was treated with MQA saturated with 0.04% MMC, applied under the conjunctiva and over the scleral flap site for 5 minutes during glaucoma filtration surgery. After surgery, 0.01% colchicine dissolved in saline (50 μL) was instilled 3 times daily.

Instillation of all types of eye drops was performed on days 1 to 14 in experiment A and on days 6 to 14 in experiment B.

### IOP measurement

IOP was measured with a pneumatonograph (Model 30 Classic Pneumatonometer; Reichert Technologies, Depew, NY, USA) after general anesthesia was induced with the intramuscular injection of a mixture of ketamine (40 mg/kg body weight) and xylazine (4 mg/kg body weight). Binocular IOP was measured within 7 min of the injection of the anesthetic. For corneal anesthesia, an oxybuprocaine solution (Benoxil ophthalmic solution 0.4%; Santen Pharmaceutical Co., Ltd.) was applied topically prior to IOP measurement. IOP measurement was performed between 3 pm and 7 pm. IOP was measured in experiment A 7, 14, 21, and 28 days after surgery. IOP was measured in experiment B 3, 7, 14, 21, 28, 35, 42, and 49 days after the surgery.

### Bleb evaluation

The blebs were examined via slit lamp and were graded with a slightly modified version of the method described by Perkins et al[19]. This method uses a qualitative scale of 1+ to 4+, reflecting increasing bleb height and size as follows: 1+, minimal height, conjunctiva thickening, no microcysts; 2+, microcysts present covering less than 75° of the eye; 3+, elevated bleb covering 75 to 135° of the eye; and 4+, greatly elevated bleb covering more than 135° of the eye. A score of 0 indicated no observable bleb. In experiment A, bleb evaluation was performed 7, 14, 21, and 28 days after surgery. In experiment B, bleb evaluation was performed 3, 7, 14, 21, 28, 35, 42, and 49 days after the surgery.

### Statistical analysis

We compared the IOP data in the treated eyes in all groups separately (two groups in experiment A: 0.04% MMC and 0.01% colchicine; and three groups in experiment B: saline, 0.04% MMC, and 0.04% MMC plus 0.01% colchicine). Values were compared with the unpaired Student’s t-test in experiment A and with the Tukey-Kramer test for multiple comparisons in experiment B (P < 0.05 was considered to be statistically significant). We also confirmed changes in slit lamp appearance and compared bleb score in these same groups with the Wilcoxon-Mann-Whitney test in experiment A and with the Steel-Dwass test for multiple comparisons in experiment B (P < 0.05 was considered to be statistically significant).

## Results

### Ocular irritation caused by colchicine

To determine the maximum safe concentration of colchicine for topical administration, the Draize test was performed. Table 1 shows the main results for total score; a detailed profile of the test results is shown in S1 Table. Eyes treated with 0.001% colchicine showed no obvious ocular irritation compared to the eyes treated with saline. The colchicine eyes had total scores of 0.0 + 0.0 at all observed time points. The eyes treated with 0.01% colchicine also showed very little ocular irritation, with total scores of 0.0 + 0.0, 0.0 + 0.0, 0.0 + 0.0, 0.0 + 0.0, 0.7 + 0.7 and 0.7 + 0.7 (n = 3, mean + S.E.M) at each observed time point (Table 1). On the other hand, the eyes treated with 0.1% colchicine showed redness in the palpebral conjunctiva and chemosis of the conjunctiva after treatment (S1 Table), with total scores of 0.0 + 0.0, 0.0 + 0.0, 0.7 + 0.7, 5.3 + 2.7, 8.0 + 2.1 and 1.3 + 0.7 (n = 3, mean + S.E.M) at each time point (Table 1).

**Table 1:** Total score of Draize ocular irritation test after treatment with colchicine.

		Total score					
	Time after the final application	0 hr	0.5 hr	1 hr	2 hr	4 hr	24 hr
Treatment	Saline	0.0 + 0.0	0.0 + 0.0	0.0 + 0.0	0.0 + 0.0	0.0 + 0.0	0.0 + 0.0
	0.1% Colchicine	0.0 + 0.0	0.0 + 0.0	0.7 + 0.7	5.3 + 2.7	8.0 + 2.1	1.3 + 0.7
	0.01% Colchicine	0.0 + 0.0	0.0 + 0.0	0.0 + 0.0	0.0 + 0.0	0.7 + 0.7	0.7 + 0.7
	0.001% Colchicine	0.0 + 0.0	0.0 + 0.0	0.0 + 0.0	0.0 + 0.0	0.0 + 0.0	0.0 + 0.0

Data represent the mean score + S.E.M. of 3 eyes for the saline group and colchicine groups (0.1%, 0.01%, and 0.001%).

These results suggest that there is a relationship between colchicine concentration and a number of key indices of ocular irritation. Minimal irritation effects were observed at a concentration of colchicine of 0.01% or less.

### Effect of colchicine and MMC on bleb preservation in a rabbit model

Firstly, we evaluated the bleb preservation effect of uncombined colchicine. We found that topical 0.01% colchicine instillation alone was less effective than the intraoperative application of 0.04% MMC in preserving reduced IOP and bleb score (S1 Fig).

**Fig 1:**
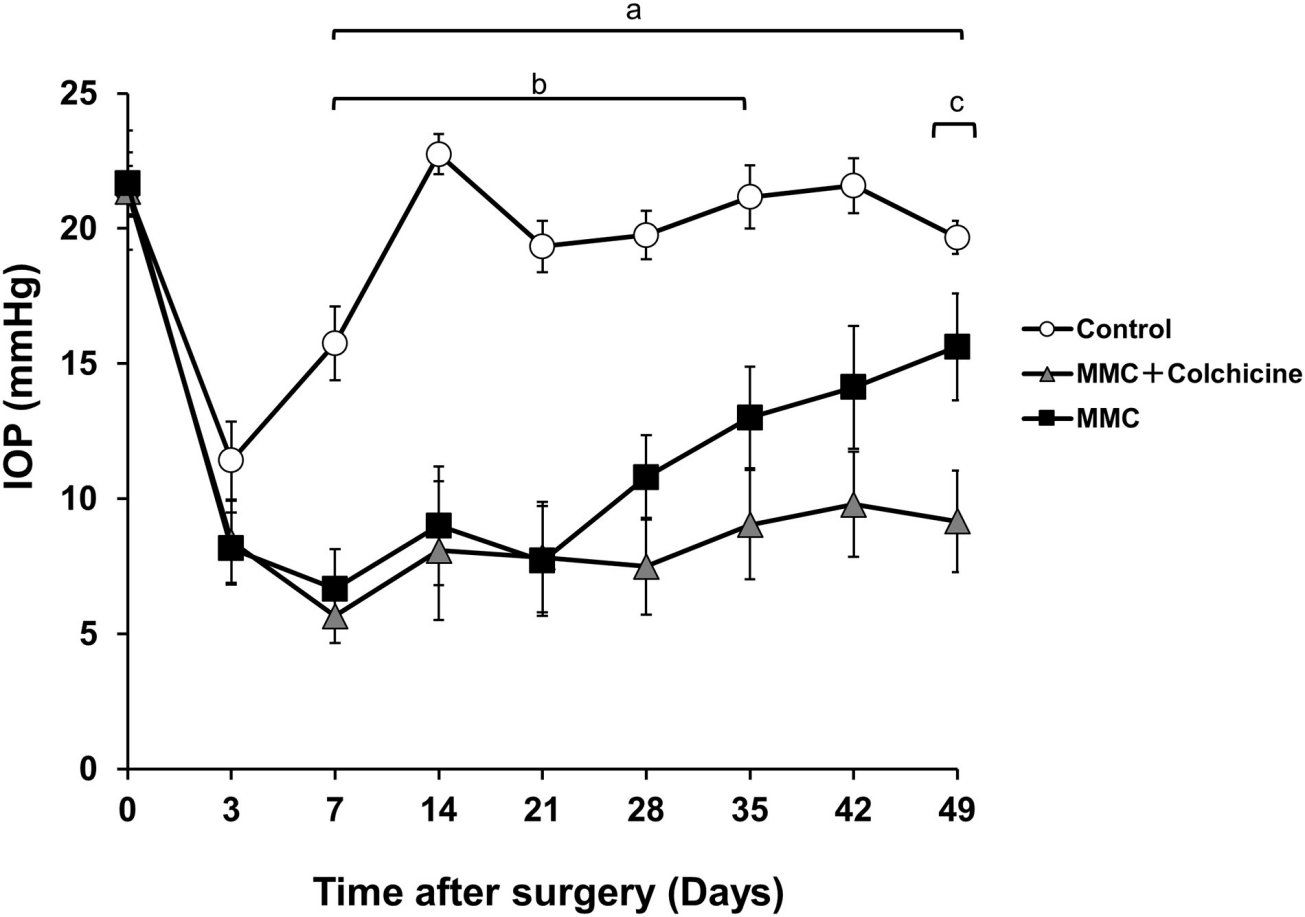
Changes in intraocular pressure (IOP) after treatment with MMC and MMC plus colchicine in a rabbit model of trabeculectomy. The white circles, black squares and gray triangles indicate controls (saline), 0.04% MMC and 0.04% MMC plus 0.01% colchicine, respectively (n = 4, 8 and 8, respectively). Error bar = SEM. The bars indicate periods with a significant difference between groups. a: 0.04% MMC plus 0.01% colchicine vs. control. b: 0.04% MMC vs. control. c: 0.04% MMC plus 0.01% colchicine vs. 0.04% MMC.

Secondly, we evaluated whether combined treatment with colchicine and MMC had a synergistic effect on bleb preservation. Fig 1 shows IOP at each measured time point in each group: control, 0.04% MMC, or 0.04% MMC plus 0.01% colchicine. There was no statistically significant difference in mean initial IOP in the groups (range: 21.4 mmHg to 21.7 mmHg). No abnormalities in the eyes or the general health of the animals were observed.

Compared to the saline group, there was a significant IOP reduction in the 0.04% MMC group at each time point between 7–35 days (P < 0.05 at all time points). The 0.04% MMC plus 0.01% colchicine group showed a significant reduction in comparison with the saline group at days 7–49 (P < 0.01 at all time points) and in comparison with the 0.04% MMC group at day 49 (P < 0.05).

**Fig 2:**
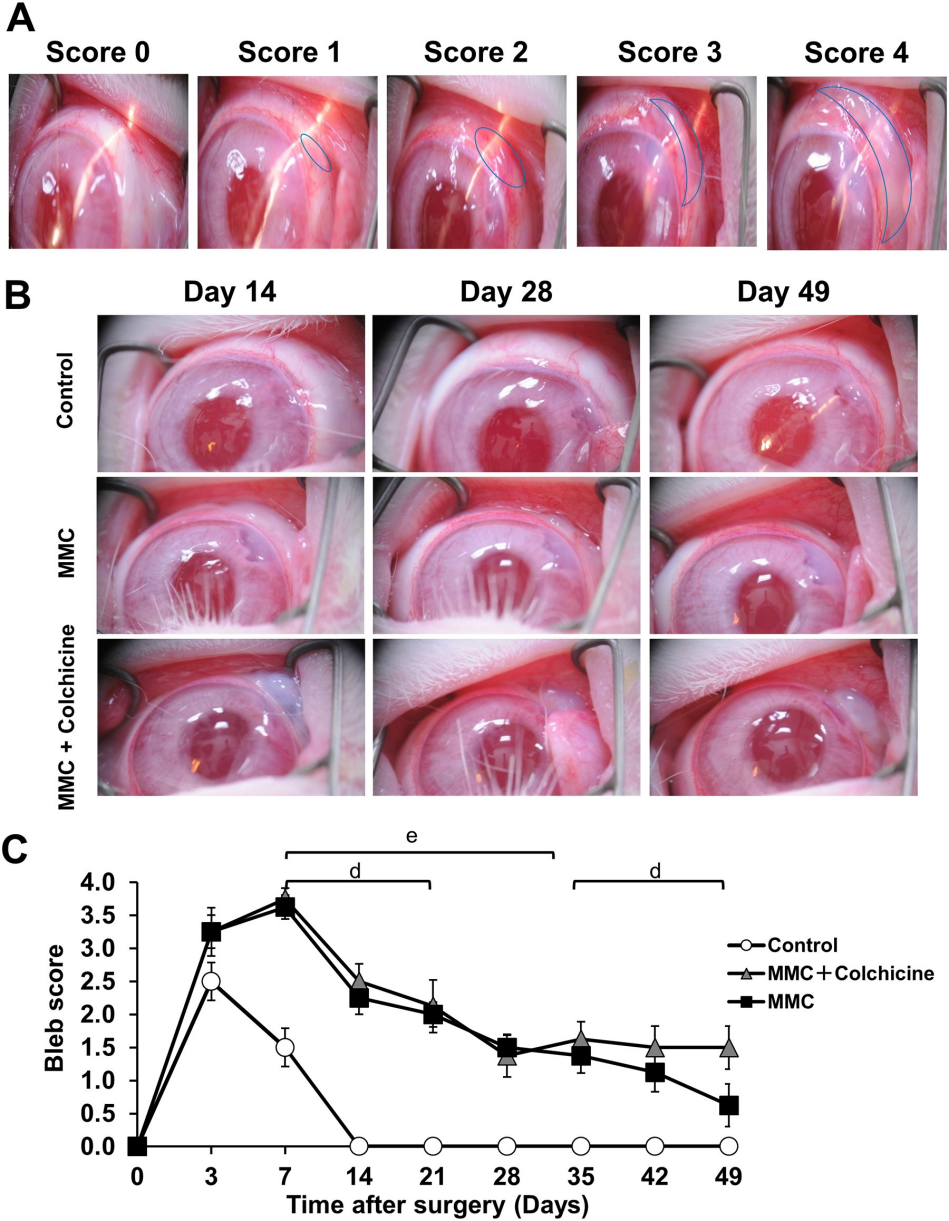
Bleb scoring via slit lamp observation. (A) Representative bleb scores (0–4) according to the method described by Perkins et al. Briefly, with increasing score, the bleb becomes more elevated and covers a wider area. (B) Representative photographs of blebs in control and treated rabbit eyes. Top to bottom rows: blebs after treatment with saline as a control, 0.04% MMC, or 0.04% MMC plus 0.01% colchicine. Left to right: images taken 14, 28 and 49 days after surgery. The blebs are visible in the upper right of each image. (C) Changes in bleb score after surgery with saline-treated controls, 0.04% MMC, and 0.04% MMC plus 0.01% colchicine. The white circles, black squares and gray triangles indicate controls, 0.04% MMC and 0.04% MMC plus 0.01% colchicine, respectively (n = 4, 8 and 8 in each group). The error bar indicates SEM. The bars indicate time periods with a significant difference between groups. d: 0.04% MMC plus 0.01% colchicine vs. control. e: 0.04% MMC vs. control.

We scored the morphology of the bleb based on its appearance and size, as previously described by Perkins TW[19]. Representative slit-lamp photographs of blebs with each score are shown in Fig 2A. Representative slit-lamp photographs of the control, 0.04% MMC, and 0.04% MMC plus 0.01% colchicine groups are shown in Fig 2B. The average bleb score of the three groups at each measured time point is shown in Fig 2C.

Compared to the saline group, the 0.04% MMC group showed a significantly higher bleb score at days 7–35 (P < 0.05 at all time points) and the 0.04% MMC plus 0.01% colchicine group showed a significantly higher bleb score at days 7–21, and 35–49 (P < 0.05 at all time points). The 0.04% MMC plus 0.01% colchicine group showed no significant difference in comparison with the 0.04% MMC group at any time point.

## Discussion

This study found that the combination of 0.04% MMC and 0.01% colchicine prolonged the low-IOP period after surgery more effectively than a control treatment. Furthermore, this period was longer than the effective low-IOP period after treatment with uncombined 0.04% MMC, in comparison to a control treatment. We also found that IOP in the 0.04% MMC group tended to increase starting at 28 days, even though the difference in IOP between the 0.04% MMC and 0.04% MMC plus 0.01% colchicine groups at 28–42 days was not statistically significant, and a statistically significant difference was only present on day 49. Similarly, combined 0.04% MMC and 0.01% colchicine preserved the subjectively-observed appearance of the bleb more effectively than in the control group. This period was longer than the similar period in the uncombined 0.04% MMC group compared to controls. The 0.04% MMC group also showed a tendency towards a worse bleb score starting at 35 days, even though there was not a statistically significant difference in bleb score between the 0.04% MMC and 0.04% MMC plus 0.01% colchicine groups at any time point. Thus, the combination of colchicine instillation and MMC application might improve the outcome of trabeculectomy by helping to maintain low IOP and a functional bleb.

MMC is used in a variety of medical settings to prevent cellular proliferation; it acts by halting the synthesis of new DNA and protein[20]. However, strong fibrosis can occur in the filtering bleb even with intraoperative MMC, causing bleb failure in some cases[7,8]. This may be because MMC-treated fibroblasts continue to express transforming growth factor-β (TGF-β), as well as pro-inflammatory cytokines such as interleukin-8 (IL-8) and monocyte chemoattractant protein-1 (MCP-1)[21,22]. These cytokines promote scarring during the remodeling phase of the wound healing cascade[23–27], and they have been observed to have mildly elevated levels in aqueous humor samples for a prolonged period after intraocular surgery[28,29].

Colchicine is considered to be an anti-mitotic drug, and acts via a very different mechanism than MMC. It is an alkaloid that binds to tubulin molecules and inhibits their polymerization into microtubules, resulting in disruption of the mitotic spindle[30], causing widespread cell death and apoptosis[31,32]. In addition, some studies have found evidence that colchicine attenuates inflammatory cytokines[33], such as TGF-β [34,35], connective-tissue growth factor (CTGF)[35], and MCP-1 [36], and suppresses extracellular matrix accumulation[36]. Other work has shown that colchicine can directly inhibit the release of fibronectin and collagen into the extracellular space by fibroblasts, reduce collagen-processing enzymes, and stimulate tissue collagenase activity[37–39]. Thus, colchicine has a broad range of anti-fibrotic functions that could complement the mechanisms underlying the ability of MMC to control scarring formation after trabeculectomy. Combined treatment with colchicine and MMC might therefore have a synergistic effect and lengthen the period of IOP reduction after trabeculectomy, in comparison to uncombined MMC.

Past studies have reported on the limitations of MMC treatment. Intraocular irrigation by the aqueous humor significantly reduces the tissue concentration of MMC, causing it to disappear rapidly in the subconjunctival tissue[40]. This may another reason that intraoperative administration of MMC alone is insufficient to keep bleb function at long postoperative period. Furthermore, inflammatory cytokines can persist for several months after surgery and counteract the anti-scarring effects of MMC[28,29,41]. These past findings suggested that new drugs might reduce scarring by suppressing this prolonged increase of inflammatory cytokines, especially if they could be applied postoperatively. Thus, previous studies investigated whether targeting specific cytokines could modulate scarring after glaucoma filtration surgery. Research in animal models showed that postoperative TGF-β inhibition with monoclonal antibodies inhibited subconjunctival scarring and prevented the failure of experimental glaucoma surgery[42]. However, clinical trials in patients undergoing primary trabeculectomy failed to reproduce this effect[43,44]. We speculated that this therapy might have failed because the monoclonal antibodies targeted only a single cytokine, TGF-β. Therefore, we hypothesized that colchicine might be more effective in modulating fibrosis after trabeculectomy, because it can suppress a variety of cytokines related to fibrosis[33–36], and can also directly suppress fibrosis by acting on fibroblasts[37–39].

Despite our finding that colchicine was effective when combined with MMC, we found that uncombined colchicine instillation was less effective than MMC in preserving bleb score and reducing IOP. This may be because a colchicine concentration of 0.01% was insufficient. Past studies of anti-metabolites as topical medicines after ocular surgery have found that these drugs must have a lower concentration when they are applied topically, rather than intraoperatively, to avoid ocular surface complications such as corneal and conjunctival damage[45,46]. Here, we found that in a Draize study, a 0.1% concentration of colchicine caused ocular irritation, so we used a 0.01% concentration, applied topically four times daily. However, interestingly, one previous study reported that the topical administration of 0.25% colchicine only once daily significantly decreased IOP up to 4 weeks after filtration surgery[47]. Thus, the possibility remains that if we adjust the number of times per day the treatment is applied, we might be able to safely increase the concentration of colchicine and improve its effectiveness.

In conclusion, this study suggests that combining topical colchicine instillation and MMC application as surgical adjuvants after trabeculectomy can increase the postoperative period during which IOP is reduced and bleb morphology is preserved. Thus, colchicine might be a promising novel adjunct to trabeculectomy, strengthening the suppression of postoperative fibrosis when used as a complement to MMC. We anticipate that this combined therapy will be explored further in future studies.

## Supporting information

S1 Table Detailed profile of Draize ocular irritation test. (XLSX)

S1 Fig Intraocular pressure (IOP) and bleb score with MMC or uncombined colchicine. The black squares and gray triangles indicate 0.04% MMC and uncombined 0.01% colchicine, respectively (all n = 4). Error bars = SEM. A: IOP; B: bleb score. The bars indicate time periods with a significant difference between groups; a: 0.04% MMC vs. uncombined 0.01% colchicine; b: 0.04% MMC vs. uncombined 0.01% colchicine.
(TIF)

S1 File Data underlying Table 1. (XLSX)

S2 File Data underlying Fig 1. (XLSX)

S3 File Data underlying Fig 2. (XLSX)

S4 File Data underlying S1 Table. (XLSX)

S5 File Data underlying S1A Fig. (XLSX)

S6 File Data underlying S1B Fig. (XLSX)
