# Supplementary figures and images for: The effectiveness of colchicine combined with mitomycin C to prolong bleb function in trabeculectomy in rabbits
Source: PLoS One. 2019 Mar 19;14(3):e0213811. doi: 10.1371/journal.pone.0213811 (PMC6424470; doi:10.1371/journal.pone.0213811)

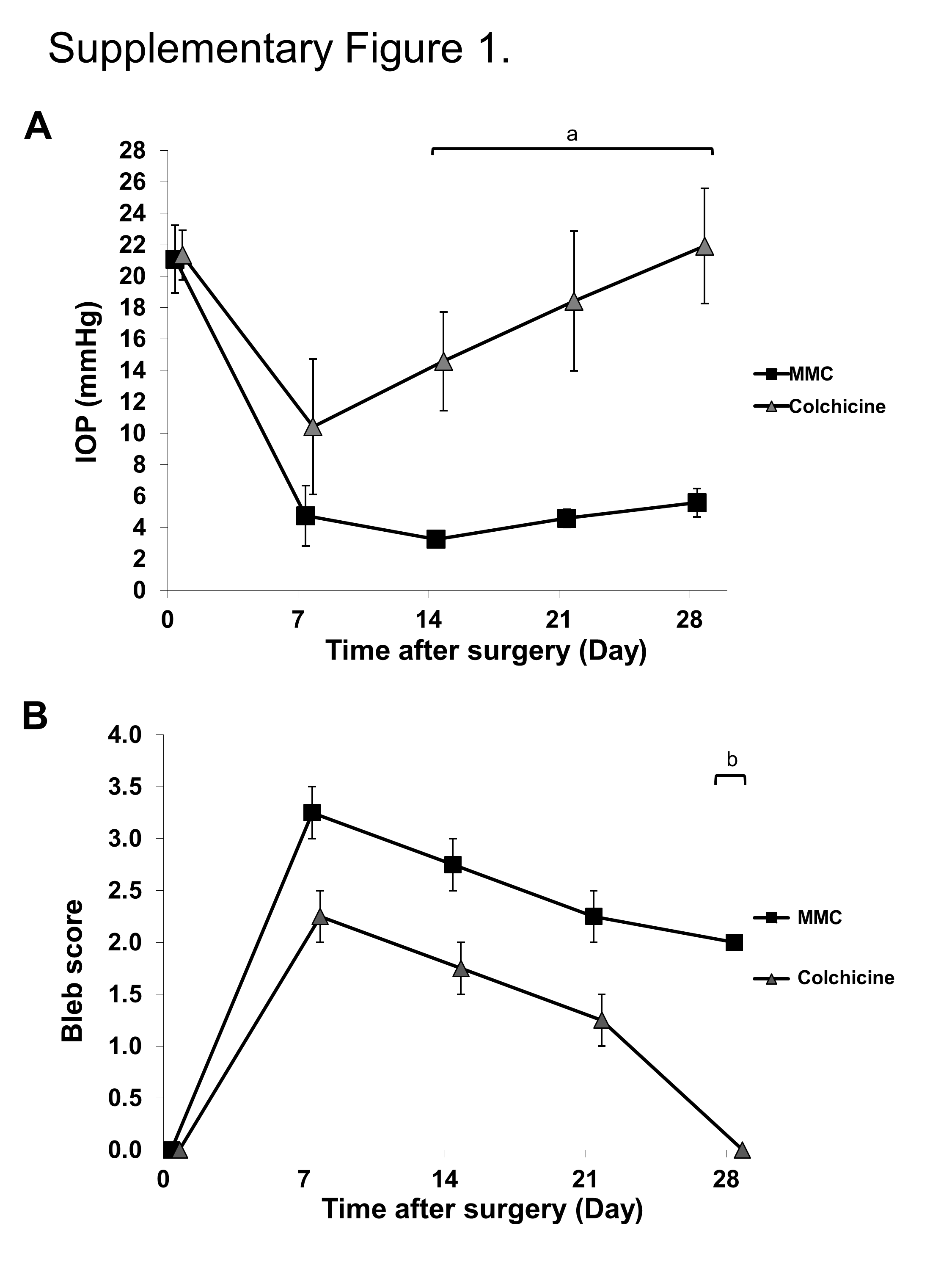

Supplement: S1 Fig — The black squares and gray triangles indicate 0.04% MMC and uncombined 0.01% colchicine, respectively (all n = 4). Error bars = SEM. A: IOP; B: bleb score. The bars indicate time periods with a significant difference between groups; a: 0.04% MMC vs. uncombined 0.01% colchicine; b: 0.04% MMC vs. uncombined 0.01% colchicine. (TIF) [file pone.0213811.s002.tif]
